# Static Body Weight Distribution and Girth Measurements Over Time in Dogs After Acute Thoracolumbar Intervertebral Disc Extrusion

**DOI:** 10.3389/fvets.2022.877402

**Published:** 2022-04-04

**Authors:** Natalia P. Amaral Marrero, Stephanie A. Thomovsky, Jessica E. Linder, Jessica Bowditch, Mallory Lind, Kristine A. Kazmierczak, George E. Moore, Melissa J. Lewis

**Affiliations:** ^1^Department of Veterinary Clinical Sciences, Purdue University College of Veterinary Medicine, West Lafayette, IN, United States; ^2^Department of Veterinary Administration, College of Veterinary Medicine, Purdue University, West Lafayette, IN, United States

**Keywords:** canine, disc herniation, spinal cord injury, digital scales, chondrodystrophic, body weight

## Abstract

Dogs with thoracolumbar intervertebral disc extrusion (TL-IVDE) can exhibit variable neurologic deficits after decompressive surgery. The objectives of this study were to quantify changes in static weight distribution (SWD) and limb and body circumference over time in dogs recovering from surgery for TL-IVDE. Dogs with acute TL-IVDE were prospectively evaluated at baseline (48–72 h post-operatively), 2, 4, 8, and 12 weeks post-operatively. Commercially-available digital scales were used to measure weight distributed to the pelvic limbs (PL%) and asymmetry between left and right pelvic limbs (LRA), each expressed as a percentage of total body weight. Trunk and thigh circumference measurements were performed using a spring-loaded tape measurement device. Measurements were performed in triplicate, compared to neurologically normal small breed control dogs and analyzed for changes over time. P <0.05 was significant. Twenty-one dogs were enrolled; 18 regained ambulation and 3 did not by study completion. PL% increased from 27.6% at baseline to 30.7% at 12 weeks but remained lower than in control dogs (37%) at all time points (*p* < 0.0001), even excluding dogs still non-ambulatory at 12 weeks (*p* < 0.025). LRA was similar to the control dogs, and did not have an association with surgical side. Caudal trunk girth decreased over time to 95% of baseline (*p* = 0.0002), but this was no longer significant after accounting for reductions in body weight (*p* = 0.30). Forward shifting of body weight persisted in dogs with TL-IVDE 12 weeks after surgery even among ambulatory dogs. SWD and circumference measurements could provide additional objective measures to monitor recovery.

## Introduction

Acute spinal cord injury (SCI) secondary to thoracolumbar intervertebral disc extrusion (TL-IVDE) in dogs can result in persistent neurologic deficits, even among dogs who regain independent ambulation. Residual functional abnormalities that have been reported in some dogs include deficits in coordination of limbs or between limbs and forward shifting of the center of pressure (1–4).

Digital scales have been used to evaluate pelvic limb static weight distribution (SWD) in dogs (5–7). In neurologically normal, chondrodystrophic small breed dogs, a mean of 63% of total body weight was borne by the thoracic limbs with a mean of 37% on the pelvic limbs (5), which is comparable to healthy large breed dogs in which 64% of body weight was placed on the thoracic limbs (7). This method was noted to be reliable and useful in dogs with osteoarthritis undergoing rehabilitation (6) but has not been explored in dogs with TL-IVDE.

Pelvic limb circumference measurements have also been reported in dogs (8–12). Thigh girth measurements were noted to be a potentially useful outcome measure in dogs with stifle disease (8) though reliability was highlighted as an issue and underscored the need to perform measurements under standardized conditions (8, 9, 11). In Dachshunds with acute TL-IVDE, thigh girth measurements were not associated with the severity of pelvic limb weakness, though measurements were only performed once at the time of initial presentation (12). When dogs with TL-IVDE were evaluated for 6 weeks post-operatively as part of a randomized clinical trial investigating rehabilitation, 80% of dogs had mild weight loss and mild decreases in thigh circumference over the first 2 weeks that largely returned to baseline by study end (13). Using a DEXA scanner in a group of Dachshunds managed surgically for TL-IVDE followed by postoperative rehabilitation, a reduction in body weight (2.2 kg), small decrease in body fat (2.4%) and increased lean muscle mass (3%) were demonstrated by 12 weeks postoperatively compared to baseline, though the majority of the dogs enrolled were overweight and specific girth measurements were not performed (14).

The SWD between thoracic and pelvic limbs and left and right pelvic limbs is unknown in dogs after acute TL-IVDE. Furthermore, it is also unknown how weight distribution changes over time, how this relates to recovery of ambulation and how this impacts musculoskeletal changes after SCI. It is possible that alterations in weight distribution could reflect and exacerbate residual pelvic limb weakness after severe SCI. Measuring such changes over time might capture persistent abnormalities and provide objective rehabilitation targets to enhance overall recovery and guide rehabilitation practices and recommendations during the post operative recovery period.

The aims of this study were: to quantify SWD and pelvic limb and trunk circumference longitudinally in dogs treated surgically for acute TL-IVDE. We hypothesized that dogs with TL-IVDE would show an initial forward shifting of body weight and increased asymmetry between left and right pelvic limbs when compared to a similar population of healthy control dogs. We further hypothesized that SWD would normalize as motor function improved and that muscle mass loss would be minimal during the study period.

## Materials and Methods

### Study Animals

Dogs with acute TL-IVDE were prospectively recruited from the existing patient pool of Purdue University Veterinary Hospital. To be included, dogs had to weigh ≤ 20 kgs, be between 1 and 10 years old, and be diagnosed with acute TL-IVDE (spinal segments T3-L3) leading to non-ambulatory paraparesis or paraplegia with or without pain perception. Duration from the onset of neurological deficits to enrollment had to be ≤ 7 days. All dogs were diagnosed via computed tomorgraphy or magnetic resonance imaging, treated with decompressive hemilaminectomy and managed post-operatively at the discretion of the clinician in charge. This included basic post-operative rehabilitation exercises during hospitalization in all dogs. Dogs with concurrent orthopedic conditons were excluded. Owners gave informed consent, and procedures were conducted in accordance to Purdue Institutional Animal Care and Use Committee (protocol #1804001743). Study dogs were compared to a previously published group of neurologically and orthopedically normal adult chondrodystrophic dogs used to develop our methods (5).

### Study Procedures

The procedures outlined were performed at baseline and at re-check visits at 2, 4, 8, and 12 weeks after initial hospitalization. The baseline visit occurred during initial hospitalization between 48 and 72 h after surgery. This ensured dogs no longer needed intravenous pain medication and increased the likelihood of being amenable to handling and successful data acquisition. All follow up visits were performed on an outpatient basis. All owners were instructed to peform daily basic rehabilitation exercises at home for the first 4–6 weeks following discharge, as is standard of care at our hospital. These included passive range of motion and massage, assisted standing and assisted walking. Owners could elect to participate in additional outpatient rehabilitation, but this was not specifically recommended and no specific instructions or goals of such therapy were provided.

### Standard Neurologic Examination and Gait Evaluation

Dogs underwent complete neurological examination including evaluation of gait, proprioception, spinal reflexes and pain perception. Dogs were videotaped walking on a flat, non-slip surface with gait deficits scored using a validated open field scale (OFS) ranging from 0 to 12 (15, 16) by two authors (NAM, MJL). Scores of 7 and above reflect the ability to take weight bearing steps 100% of the time. Using OFS scores, dogs were classified as high-functioning (HF, OFS ≥ 7 by 12 weeks or sooner) or low-functioning (LF, OFS <7 by 12 weeks).

### Body Weight Distribution

Static weight distribution was determined using commercially available, factory-calibrated digital bathroom^1^ (range 1.4 to 200 kg, 0.1 kg accuracy) or kitchen scales^2^ (range 1 g to 5 kg, 1 g accuracy) as previously described (5). Briefly, dogs were placed in a standing position, with each limb centered on an individual scale or, separately, with each pair of limbs (thoracic or pelvic) centered on a scale (Supplementary Figures 1, 2). A trial was considered successful when a dog stood still and unassisted for at least 3 seconds, with their head facing forward. Dogs were allowed to acclimate to standing on the scales for several minutes before measurements began. This included being able to explore the scales, interact with the handlers and being placed in a standing position several times. Testing was then performed in a quiet environment free from distractions. Dogs unable or unwilling to stand unassisted were not included in analysis for that method at that timepoint. All scale measurements were acquired in kilograms and performed in triplicate with brief breaks between each acquisition.

Using two bathroom scales, pelvic limb SWD (PL%) was defined as the weight borne by the pelvic limbs as a percentage of total body weight. Using four kitchen scales, the weight distributed to left or right pelvic limbs was also expressed as a percentage of total body weight (LH% or RH%, respectively). For dogs above the weight range for the kitchen scales (>5 kg for an individual limb), four bathroom scales were used to capture data on left and right limbs individually. Left to right asymmetry (LRA) of pelvic limbs was defined as the difference between RH% and LH%. For a given visit, the following definitions of LRA were utilized:

- Values ≥ 5% were considered “leaning right” (i.e. bearing more weight on the RH),
- Values ≤ −5% were considered “leaning left” (i.e. bearing more weight on the LH)
- Values between −5% and 5% were categorized as “no lean”

Dogs were then classified overall as follows:

- “Leaning right” if LRA exceeded 5% on at least one visit
- “Leaning left” if LRA was < -5% on at least one visit
- “No lean” if LRA was between −5 and +5% for all visits
- “Both” if LRA was >5% and < -5% on separate visits

To evaluate asymmetry between pelvic limbs irrespective of direction, absolute LRA (aLRA) was defined as the absolute value of the LRA between pelvic limbs.

### Body and Limb Circumference Measurements

Using a Gulick type II spring-loaded tape measurement device^3^, right and left thigh, cranial trunk, and caudal trunk girth measurements were performed (Supplementary Figure 3). While dogs were lying in lateral recumbency, hind limb circumference of the upper leg was measured at 50% of the thigh length from the greater trochanter to the distal femur (12, 17). Trunk measurements were performed in a standing position. Cranial trunk girth was measured around the rib cage, immediately caudal to the thoracic limbs, while caudal trunk girth was measured around the abdomen just cranial to the inguinal folds. All measurements were made in triplicate by one of two authors (JB or ST). To account for variations in size and weight between dogs, all circumference measurements for follow up visits were expressed as a percentage of the baseline value (100%).

### Statistical Analysis

Summary statistics are reported as mean (standard deviation) or median (range), for parametrically or nonparametrically distributed data respectively, based on a Shapiro-Wilk test for normality. Analysis for statistical significance was performed using SAS PROC MIXED mixed linear effects model for repeated measures with *post-hoc* Bonferroni adjustment for multiple comparisons; statistical significance was defined as *p* < 0.05.

Mean PL% of TL-IVDE dogs at each visit was compared to mean PL% for the healthy control dogs (5). Excluding the LF dogs (*n* = 3, OFS <7 by 12 weeks) who did not recover ambulation in the typical timeframe, PL% of the HF dogs at each visit was also compared to PL% for the control dogs. For all TL-IVDE dogs, changes in PL% over time were assessed in the mixed effects model with dog as a random effect. Pearson correlation coefficient was calculated to evaluate the relationship between PL% and OFS at each visit.

A Fisher's exact test was performed to evaluate the association between dogs classified as leaning right or leaning left and the side of hemilaminectomy (right or left). Dogs categorized as “no lean” or “both” (i.e. “leaning left” and “leaning right” on separate visits), were excluded from the analysis. Absolute LRA was compared between the control dogs and TL-IVDE dogs for each visit, assessing changes in asymmetry of pelvic limbs over time.

Circumference of left and right pelvic limbs, cranial trunk girth, and caudal trunk girth were analyzed for changes over time using analysis with baseline data compared to follow up visits. To account for changes in overall body weight over time, measurements were expressed as a ratio by dividing by total body weight.

## Results

### Study Population

Twenty one dogs were enrolled in the study: nine Dachshunds, two Maltese, one Pekingese, one Lhasa Apso, one French Bulldog, one Cocker-Spaniel, and six chondrodystrophic mixed breed dogs. There were eleven males and ten females, with a mean age of 5.4 years (SD 2.4) and mean body weight of 7.4 kg (SD 2.7). The mean body weight of the TL-IVDE dogs in this study was significantly lower than the healthy control dogs used for comparison (mean body weight: 12.1 kg, SD 3.28, *p* < 0.0001), though the age and breed distribution were similar (5). At baseline, 14 dogs were non-ambulatory paraparetic, four were paraplegic with intact pain perception, and 3 were paraplegic without pain perception.

The mean time interval between the onset of neurological signs to decompressive surgery was 38 h (SD 20), and the mean time interval from onset of neurological signs to enrollment in the study was 4 days (SD 1). Of the 21 dogs, 16 completed all visits. Twenty dogs completed a 2 week visit (±5d), 20 dogs had a 4 week visit (±5d), 17 dogs had an 8 week visit (±5d), and 18 dogs had a 12 week visit (±14d). All dogs participated in basic post-operative rehabilitation during initial hospitalization and were instructed to continue to perform passive range of motion, massage, assisted standing and assisted walking at home until they were walking or the 4-week recheck. Two dogs participated in outpatient rehabilitation programs (at other hospitals) and specific details or exercise regimens were not available.

### Gait Scoring

Table 1 outlines the OFS for each study visit. Eighteen dogs were categorized as HF (OFS score of ≥ 7 by 12 weeks or sooner); 10/18 achieved this score by 2 weeks, 5/18 by 4 weeks, and 3/18 by 8 weeks. Three dogs did not recover ambulation by 12 weeks and were designated as LF. Two of the LF dogs were initially deep pain negative and did not regain pain perception in the duration of the study, while the third was initially paraplegic with blunted but intact pain perception.

**Table 1:** Gait scores for each study visit.

**Study visit**	**Median (range) OFS**		
	**All dogs**	**HF dogs**	**LF dogs**
Baseline	2 (0–6)	2 (0–6)	0 (0)
2 week	6 (0–9)	7 (2–9)	0 (0–1)
4 week	9 (1–12)	9 (5–12)	1 (1–3)
8 week	9 (1–11)	9 (7–12)	2 (1–4)
12 week	10 (1–12)	11 (8–12)	2 (1–5)

*OFS, open field score (0–12); HF, high functioning group (OFS ≥ 7 by 12 weeks or sooner), LF, low functioning group (OFS <7 by 12 weeks)*.

### Pelvic Limb Static Weight Distribution

**Figure 1:**
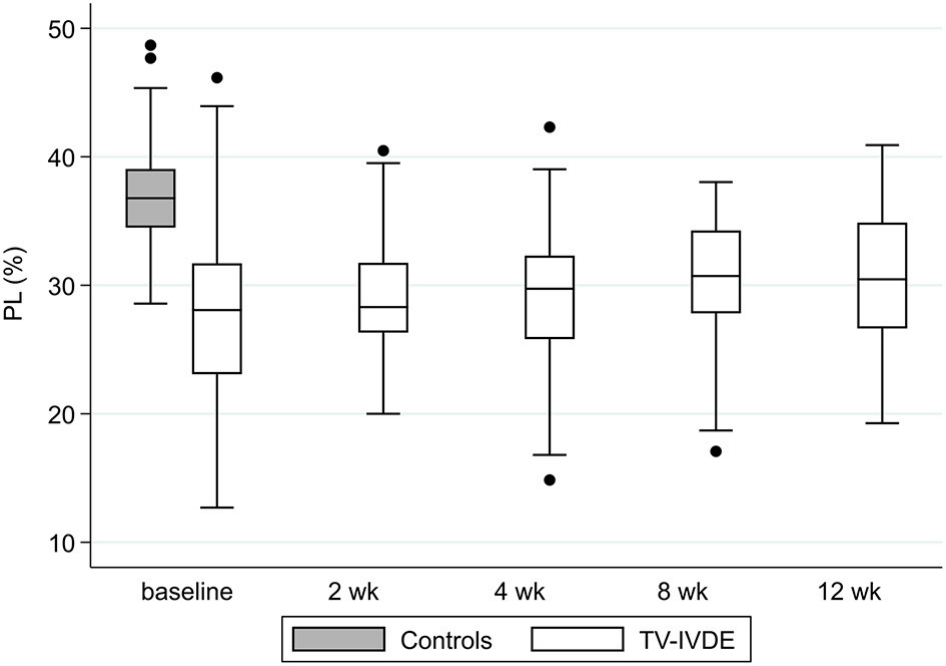
PL% in normal and TL-IVDE dogs at baseline and across study visits. PL%: pelvic limb static weight distribution as a percentage of total body weight.

Mean PL% at each visit is summarized in Table 2 and Figure 1. Three dogs were unable to meet the definition of standing at baseline (2 LF, 1 HF), and 1 of these LF dogs was also unable to stand at the 2, 4 and 8 week visits. These dogs were not included in summary data for these visits. Values generally increased over time ranging from 27.6 to 30.7% but the differences we not significant after adjusting for multiple comparisons (*p* = 0.26). Compared to the mean PL% of 37% reported in the control dogs (5), the mean PL% of all TL-IVDE dogs was significantly lower at all timepoints (*p* < 0.0001). When just considering the HF group, PL% was still significantly lower than in the control dogs at each visit (*p* < 0.025). Open field scores and PL% were moderately correlated at each visit (r = 0.50, r^2^ = 0.25, *p* < 0.0001).

**Table 2:** Pelvic limb static weight distribution as a percent of total body weight (PL%) at each study visit.

**Study visit**	**Mean (SD) PL%**		
	**All dogs**	**HF dogs**	**LF dogs**
Baseline	27.6 (6.5)	27.3 (6.6)	NA
2 week	29.1 (4.6)	29.4 (4.7)	26.4 (1.0) (*n* = 2)
4 week	29.0 (5.4)	29.2 (5.6)	26.9 (2.5) (*n* = 2)
8 week	30.1 (5.2)	30.3 (5.5)	28.8 (1.2) (*n* = 2)
12 week	30.7 (5.1)	30.7 (5.0)	30.8 (7.4) (*n* = 3)

*HF, high functioning group; LF, low functioning group*.

### Asymmetry Between Left and Right Pelvic Limbs

Eleven dogs had LRA ≥ 5% were classified as “leaning right.” In 7/11 dogs this was noted at a single timepoint while 4 dogs were noted to lean to the right at 2 to 4 study visits. Two dogs had LRA ≤ -5% and were classified as “leaning left,” both of which leaned to the left on 3 occasions. Four dogs were classified as “no lean,” 3 dogs had LRA values < −5% and >5%, and individual limb data was not available in one dog precluding determination of LRA. Eleven of the 13 dogs (85%) that were classified as leaning left or right during their recovery bore more weight on the pelvic limb opposite from the side of surgery, though the relationship between surgery side and the direction of leaning was not significant (*p* = 0.077).

**Figure 2:**
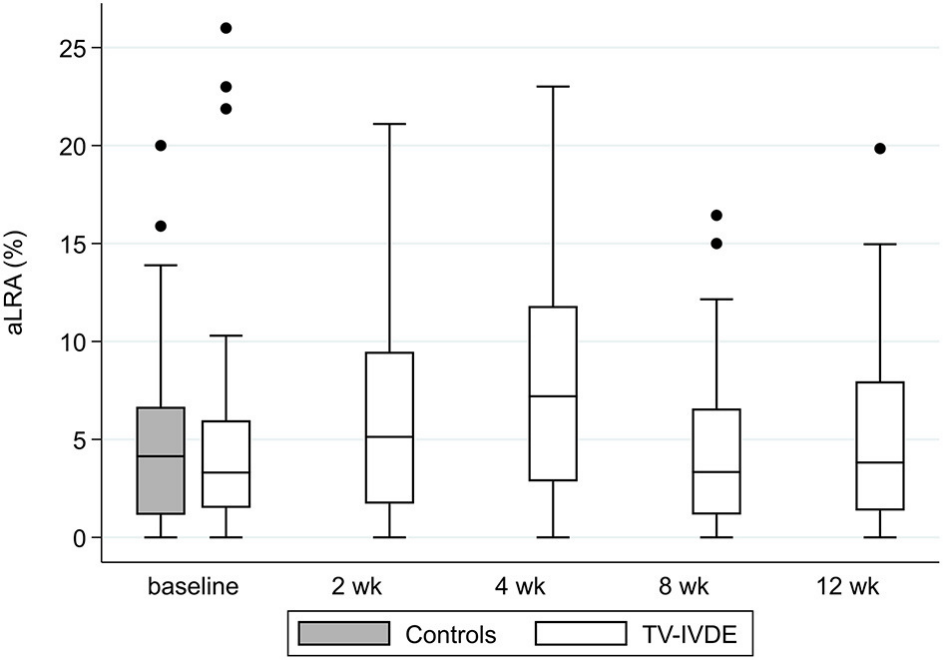
Absolute LRA for each study visit in dogs with TL-IVDE compared to healthy control dogs. aLRA: absolute left right asymmetry between left and right pelvic limbs.

Absolute left right asymmetry (aLRA) for all dogs across study visits ranged from 0.2 to 26.0%(median 5.1%), compared to a median of 4.9% (0.5–20.0%) in the healthy control dogs (Table 3; Figure 2). No significant differences were identified in aLRA between the control dogs and TL-IVDE dogs at any visit (*p* = 0.079). Among the TL-IVDE dogs, asymmetry between pelvic limbs was greatest at the 4 week visit but no significant changes over time were noted (*p* = 0.17).

**Table 3:** Absolute LRA for each study visit.

**Study visit**	**Median (range) aLRA %**
	All dogs
Baseline	4.0 (0.7–26.0)
2 week	6.0 (0.2–21.1)
4 week	7.6 (1.0–23.0)
8 week	4.8 (0.7–16.4)
12 week	4.9 (0.8–19.8)

*aLRA, absolute left right asymmetry between left and right pelvic limbs*.

### Body and Limb Circumference Measurements

**Figure 3:**
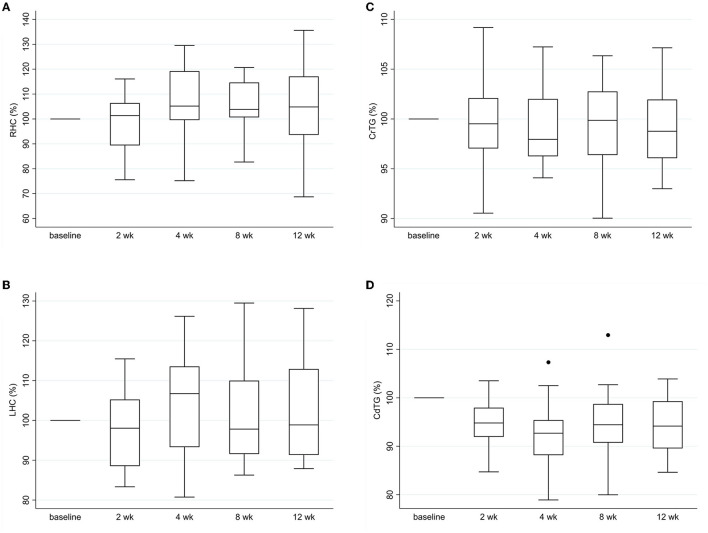
Trunk and limb circumference measurements over time. **(A)** right thigh girth, **(B)** left thigh girth, **(C)** cranial trunk girth and **(D)** caudal trunk girth for each study visit as a percentage of baseline values. RHC: right hind limb circumference, LHC: left hind limb circumference, CrTG: cranial trunk girth, CdTG: caudal trunk girth.

Limb and trunk circumference measurements are summarized in Table 4. Mean right and left thigh circumference as a percentage of baseline measurements showed a slight decrease at 2 weeks but were the same to mildly higher than baseline values at all other study visits (Figures 3A,B). Changes over time for right and left thigh circumference were not significant (*p* = 0.0821, *p* = 0.29). Cranial trunk girth as a percentage of baseline measurements demonstrated minimal change across study visits (*p* = 0.95) (Figure 3C). Caudal trunk girth significantly decreased over time (*p* = 0.0002) with the greatest decrease at the 4 week visit of nearly 8% (Figure 3D). Compared to baseline, caudal trunk girth was significantly decreased at weeks 2, 4 and 12 weeks (*p* < 0.038). Caudal trunk girth expressed as a ratio of total body weight was still lower at follow up visits compared to baseline but the differences were no longer significant (*p* = 0.30).

**Table 4:** Mean thigh and body circumference measurements as a percentage of baseline values.

**Study visit**	**Left thigh girth % (SD)**	**Right thigh girth % (SD)**	**Cranial trunk girth % (SD)**	**Caudal trunk girth % (SD)**	**Caudal trunk girth to body weight % (SD)**
Baseline	100	100	100	100	100
2 week	97.7 (9.0)	97.7 (11.5)	99.8 (4.1)	95 (4.8)	98.2 (4.4)
4 week	103.9 (13.5)	106.9 (15.8)	99.2 (3.9)	92.8 (7.1)	96.8 (4.6)
8 week	100.6 (12.0)	103.5 (12.0)	99.4 (4.4)	95 (7.6)	98.3 (6.2)
12 week	103.6 (13.8)	105 (16.8)	99.3 (3.8)	94.4 (6.0)	98.2 (5.3)

## Discussion

Dogs with acute TL-IVDE treated with decompressive surgery demonstrated altered body weight distribution during the recovery period. While weight distributed to their pelvic limbs increased over time, it did not normalize by 3 months post-operatively when compared with values in neurologically normal, chondrodystrophic small breed dogs. Changes in girth measurements during the study period were small and impacted by variability, but forward shifting of weight could have contributed to reductions in hindquarter muscle mass. Asymmetry in weight distribution between left and right pelvic limbs was similar to asymmetry present in normal dogs and lacked clear trends over time.

Our results demonstrated that dogs with acute TL-IVDE managed surgically leaned forward during their recovery. Compared to a group of neurologically normal, healthy small breed dogs that bore 37% of total body weight on their pelvic limbs, the dogs of this study bore an average of <31% on their pelvic limbs over the first 3 months post-operatively. The dogs of this study likely persistently leaned forward to compensate for ongoing pelvic limb weakness. This is consistent with a more cranial location of the center of pressure reported in dogs with SCI secondary to TL-IVDE (2, 3). Abnormal, compensatory shifts in static weight distribution have also been reported in dogs with pelvic limb osteoarthritis (6) and in experimentally-induced pelvic limb lameness in dogs (18). Forward shifting of body weight after TL-IVDE might be an expected finding among the dogs that remained non-ambulatory by study end. However, contrary to what we anticipated, even dogs that were strongly ambulatory still bore less weight on their pelvic limbs 3 months after injury and surgery as compared to normal controls. While we did not evaluate long-term outcomes, our findings support that altered weight distribution can persist in the short-term after TL-IVDE managed surgically.

Approximately 60% of the dogs in this study showed a >5% discrepancy in weight distributed between left and right pelvic limbs. While 85% of these bore more weight on the leg opposite the side of surgery, this relationship was not signfiicant, likely due to low numbers. Asymmetry of neurologic deficits between the pelvic limbs in dogs with TL-IVDE is common with the more severely affected limb typically corresponding to the side of greater compression and therefore the side of decompressive hemilaminectomy (19). This is also consistent with dogs with lameness secondary to stifle disease where dogs leaned away from the lame limb (18). However, of the 13 dogs designated as leaning, 7 leaned beyond the 5% cutoff at only a single visit. Three additional dogs leaned both left and right (on different visits) and four dogs did not lean notably in either direction at any visit. Additionally, asymmetry between pelvic limbs did not decrease over time as dogs recovered and was generally comparable to that of the healthy control dogs in which is was just under 5%. These findings highlight the limitations of measuring individual limbs, which was previously noted to be challenging in a study of weight distribution in large breed dogs with and without osteoarthritis (6). External factors such as handler position, leash side and location of a wall have been shown to impact dynamic weight distribution in walking dogs (20, 21), and could also impact SWD. We required dogs to stand still and squarely, but small shifts in position likely contributed to and magnified the variability of individual limb measurements, limiting the ability to utilize individual limb changes to track recovery.

While abnormal static weight distribution, notably leaning forward, persisted by 3 months post-operatively, the clinical relevance of these findings are unknown. A successful outcome for dogs with TL-IVDE has been defined as recovery of ambulation and continence and the resolution of pain (22, 23). While this is a functionally acceptable outcome, deficits in strength and coordination can persist (1–4). Binary assessment of ambulation (yes or no) or the commonly applied modified frankel score can have a ceiling effect in evaluating functional status once ambulation is achieved and are limited to detect more nuanced aspects of recovery (24). While the vast majority regained ambulation and 13/18 (72%) had normal or nearly normal OFS scores by study end, the altered weight distribution could perpetuate or exacerbate residual weakness in one or both pelvic limbs. In people with spinal cord injury, dynamic weight shifting exercises have been shown to enhance step length and walking distance and improve overall locomotor outcomes after injury (25, 26). Building upon our preliminary results, future prospective studies in dogs could more directly evaluate the effect of stance and weight distribution on recovery of locomotion following spinal cord injury. The role of rehabilitation exercises focusing on improving weight distribution when standing and ambulating is also a needed area of study.

Beyond ambulation, the implications for long-term decrease in weight bearing have not been studied in the context of TL-IVDE, but can be extrapolated from other contexts in which a variety of musculoskeletal changes have been described. There is evidence that immobilization alters nerve function and inter-joint coordination (27, 28). Canine models for ligament damage also showed that increased muscle loading results in greater range of motion and biomechanical properties of musculoskeletal tissues after experiencing injury (29). Additionally, chronically increased weight bearing on the thoracic limbs could contribute to or exacerbate osteoarthritis of the elbow or shoulder joints, or even perpetuate neurologic dysfunction or injury in the cervical or thoracic spine. In dogs with a left to right discrepancy, leaning away from a weaker pelvic limb could abnormally increase loading on the stronger limb.

Caudal trunk girth measurements decreased over time. By 2 weeks post-operatively, they were significantly lower than baseline and remained lower throughout the study period. While this change was small, it might reflect loss of muscle mass of the caudal lumbar epaxial and gluteal muscles in recovering dogs. This could be explained by overall reductions in body weight and generalized disuse atrophy secondary to post-operative activity restrictions, but forward shifting of body weight resulting in decreased hindquarter loading could have been a contributing factor. Mean thigh girth measurements decreased mildly at 2 weeks but were increased slightly at the final study visit compared to baseline values. While significant changes in thigh circumference were not identified across study visits, these findings are consistent with prior reports in dogs with TL-IVDE and stifle disease where small initial decreases in thigh circumference were noted (8, 13). In dogs with TL-IVDE, this decrease largely returned to baseline by 6 weeks post-operatively (13) and small increases in lean muscle mass have also been noted by 3 months post-operatively (14). Importantly, thigh circumference measurements presented several challenges. Limb conformation was previously noted to be a limitation for obtaining girth measurements in dachshunds (12). This study was not limited to dachshunds, but the vast majority were chondrodystrophic and limb conformation hampered performing consistent measurements, even for trained personnel. Additionally, some dogs were more relaxed than others when laying in lateral recumbency, and limb position (flexed or extended) was not standardized. In prior studies of circumference measurements in normal dogs and dogs with musculoskeletal diseases, thigh girth was noted to have poor reliability (11) and limb position affected results (8). In comparison, caudal trunk girth was performed with dogs standing squarely. This might have resulted in fewer variations in positioning and other patient-related factors, potentially leading to more reliable results between dogs and over time.

Physical rehabilitation is commonly recommended as a routine component of post-operative care for dogs with TL-IVDE (30). Despite the high frequency, there are few validated outcome measures available to evaluate the impact of rehabilitation protocols in neurologic dogs. Objective tools are commonly utilized in dogs with orthopedic injuries to determine success of post-injury physical rehabilitation (31). Our results demonstrate that measuring SWD using digital scales can be easily incorporated into post-operative assessment. With further standardization of acquisition protocols, circumference measurements, especially caudal trunk grith, might also be useful to track changes in muscle mass. Together, they could serve as objective targets for formal or informal rehabilitation programs, providing the rehabilitation practitioner with specific ways to broadly assess recovery from establishing a starting point to gauging success over time. Such measurements will allow improved design and adaptation of of individual therapy regimens for dogs with spinal cord injury. Future prospective clinical trials could investigate if exercises to emphasize pelvic limb weight bearing (e.g., walking up small inclines or utilizing ramps or balance boards) result in improved SWD and improved locomotor outcomes, and over what timeframe and intensity such exercises are needed to produce a quantifiable benefit.

Limitations of this study include a small sample size. While the majority of dogs completed all study visits, there was a small amount of missing data at each follow-up visit which further contributed to our limited numbers and could have influenced our results. We also only enrolled a small number of severely affected dogs. Paraplegic dogs with absent pain perception due to TL-IVDE have worse outcomes compared to dogs where pain perception is maintained (23, 32). This group might be most likely to benefit from physical rehabilitation; validation of outcome measures are warranted specifically to track their recovery. Across all dogs, evaluation over a longer time period might be needed to assess if some of the observed changes normalize with additional time and, even if persistent, if such changes are functionally or clinical relevant for a given dog. Additionally, validating these measurements with other objective markers such as bone density (DEXA) scans or assessment of muscle strength might be useful to evaluate their clinical utility.

Overall, dogs recovering from acute TL-IVDE demonstrated a persistent tendency to lean forward even among dogs with minimal to no visible gait deficits. These alterations in SWD might contribute to changes in muscle mass and perpetuate residual pelvic limb weakness.

## Data Availability Statement

The raw data supporting the conclusions of this article will be made available by the authors, without undue reservation.

## Ethics Statement

The animal study was reviewed and approved by Purdue Institutional Animal Care and Use Committee (protocol #1804001743). Written informed consent was obtained from the owners for the participation of their animals in this study.

## Author Contributions

NAM participated in data analysis, manuscript preparation, and editing and review. ST, JL, and MJL participated in study design, data acquisition and analysis, manuscript preparation, and editing and review. JB, ML, and KK participated in data acquisition and analysis, and manuscript editing and review. GEM participated in data analysis with statistical support, manuscript preparation, and editing and review. All authors contributed to the article and approved the submitted version.

## Conflict of Interest

The authors declare that the research was conducted in the absence of any commercial or financial relationships that could be construed as a potential conflict of interest.

## Publisher's Note

All claims expressed in this article are solely those of the authors and do not necessarily represent those of their affiliated organizations, or those of the publisher, the editors and the reviewers. Any product that may be evaluated in this article, or claim that may be made by its manufacturer, is not guaranteed or endorsed by the publisher.

## Footnotes

^1^Smart Weight Smart Tare Digital Body Weight Bathroom Scale, amazon.com.

^2^ETEKCITY Digital Kitchen Scale, Model No EK6212-S, amazon.com.

^3^Gulick II Tape Measure, Fitness Mart, GaysMills, WI.

## Supplementary Material

Supplementary Figure 1 Two bathroom scales stance, side view (left) and top view (right).

Supplementary Figure 2 Four kitchen scales stance, side view (left) and top view (right).

Supplementary Figure 3 Body and limb circumference measurements. Top row: cranial trunk girth, cranial trunk girth close-up, thigh girth; Bottom row: caudal trunk girth, caudal trunk girth close-up.

## Abbreviations

TL-IVDE: thoracolumbar intervertebral disc extrusion
SCI: spinal cord injury
SWD: static weight distribution
OFS: open field scale
PL%: pelvic limb static weight distribution as percentage of total body weight
LRA: left right asymmetry between pelvic limbs
aLRA: absolute left right asymmetry between pelvic limbs.
