# Supplementary figures and images for: Static Body Weight Distribution and Girth Measurements Over Time in Dogs After Acute Thoracolumbar Intervertebral Disc Extrusion
Source: Front Vet Sci. 2022 Apr 4;9:877402. doi: 10.3389/fvets.2022.877402 (PMC9013748; doi:10.3389/fvets.2022.877402)

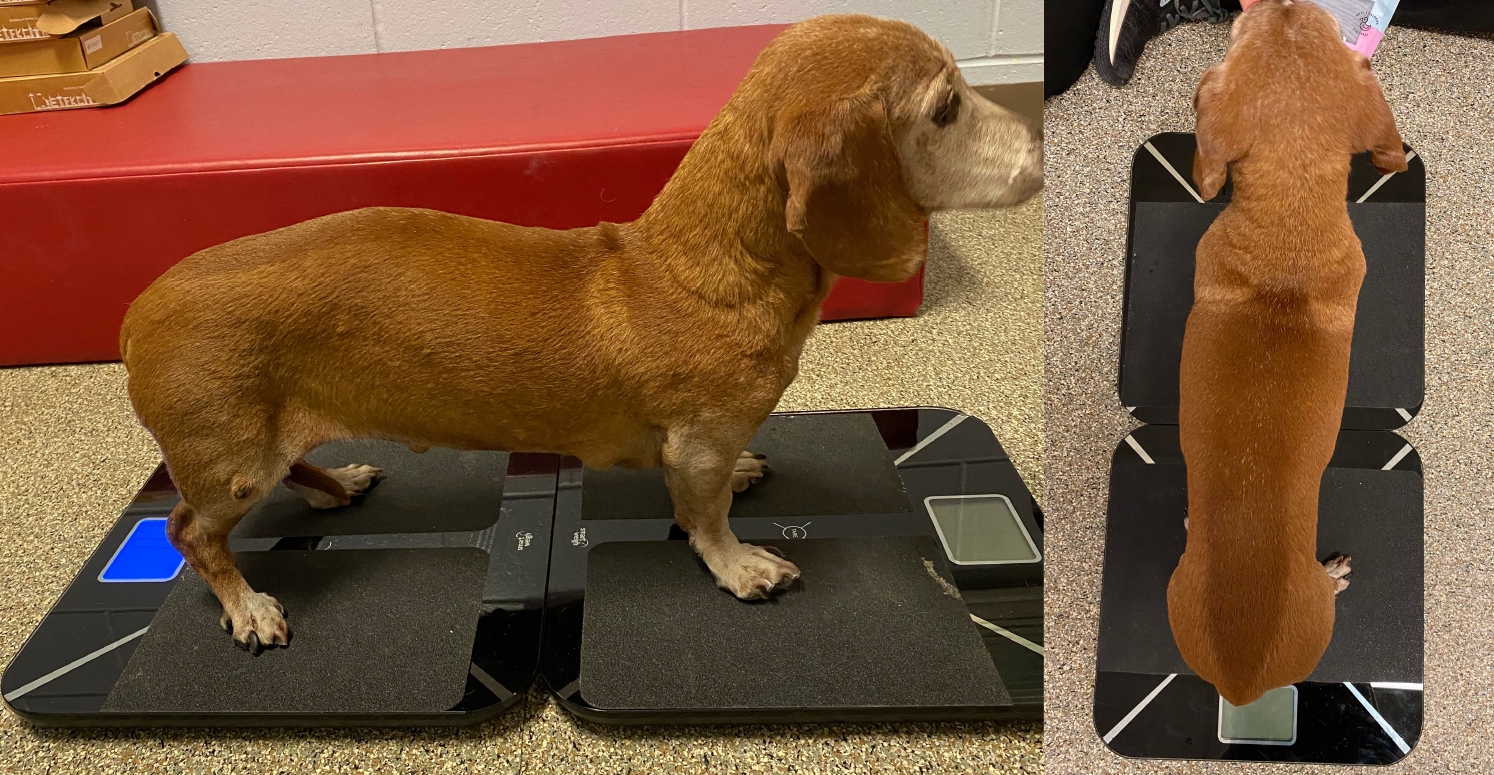

Supplement: Supplementary Figure 1 — Two bathroom scales stance, side view (left) and top view (right). [file Image_1.TIF]

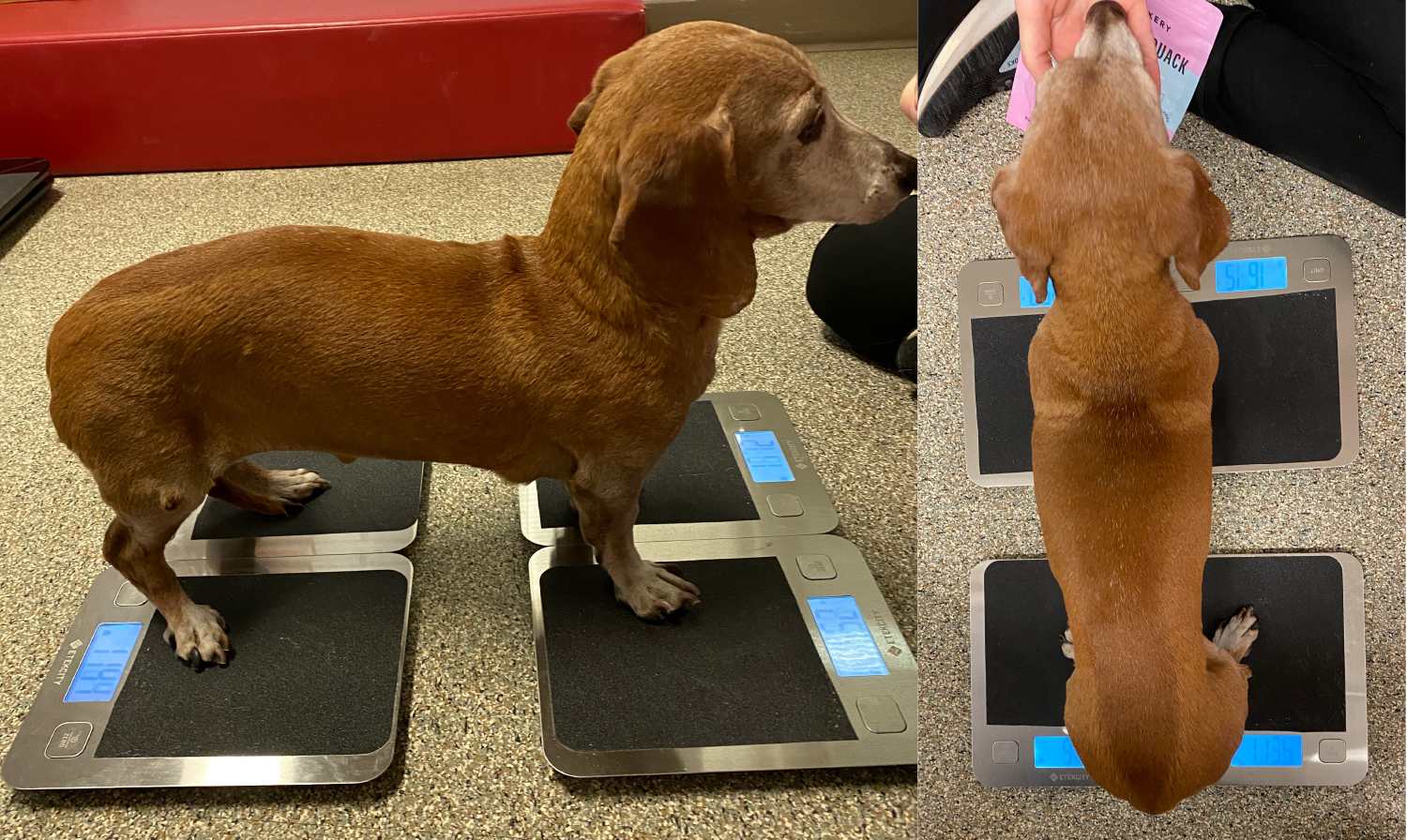

Supplement: Supplementary Figure 2 — Four kitchen scales stance, side view (left) and top view (right). [file Image_2.TIF]

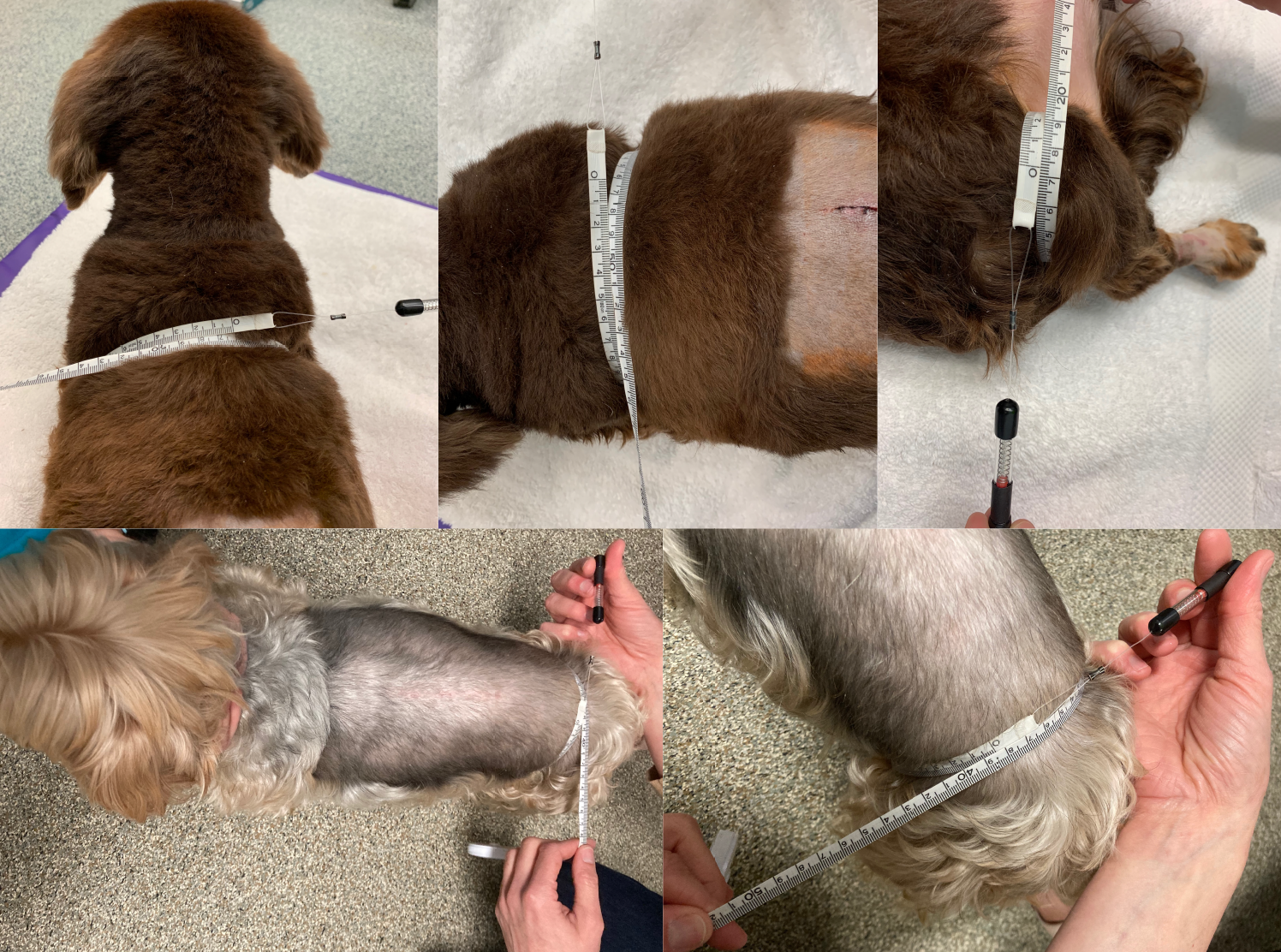

Supplement: Supplementary Figure 3 — Body and limb circumference measurements. Top row: cranial trunk girth, cranial trunk girth close-up, thigh girth; Bottom row: caudal trunk girth, caudal trunk girth close-up. [file Image_3.TIF]
